# Teaching trainee psychiatrists a Mentalization-Based Treatment approach to personality disorder: effect on attitudes

**DOI:** 10.1192/bjb.2021.50

**Published:** 2022-10

**Authors:** Tennyson Lee, Patrick Grove, Chris Garrett, Thomas Whitehurst, Orestis Kanter-Bax, Kamaldeep Bhui

**Affiliations:** 1 DeanCross Personality Disorder Service, London, UK; 2 Centre for the Understanding of Personality Disorder (CUSP), London, UK; 3 East London NHS Foundation Trust, London, UK; 4 Essex Partnership University NHS Foundation Trust, Wickford, UK; 5 University of Oxford, UK

**Keywords:** Borderline personality disorder, education and training, Mentalization-Based Treatment, attitudes to personality disorder, clinical skills

## Abstract

**Aims and method:**

To evaluate whether a brief training using a Mentalization-Based Treatment (MBT) model improves attitudes of trainee psychiatrists working with patients with personality disorder. Trainee psychiatrists (*n* = 49) completed the Attitudes to Personality Disorder Questionnaire before and after a training consisting of two 3 h lectures on (a) theory of personality disorder and (b) practical skills using an MBT role-play.

**Results:**

There was a significant improvement on composite scores of attitude, with small to moderate effect size (Wilcoxon signed-rank test *Z* = 3.961, *P* < 0.001, *r* = 0.40).

**Clinical implications:**

Brief MBT-informed teaching oriented to the clinical situation appears to have a positive effect on attitudes towards people with personality disorder.

In response to a consensus statement on personality disorder that described the neglect and exclusion of those given the diagnosis,^1^ the Royal College of Psychiatrists’ position statement on personality disorder stressed the need for staff training, supervision and reflective practice.^2^

Many clinicians have unacceptably pejorative attitudes regarding people diagnosed with a personality disorder. People with the diagnosis may be seen by psychiatrists as more difficult, manipulative and annoying.^3,4^ A borderline personality disorder (BPD) diagnosis is thought to be more difficult to deal with^5^ and can lead to more negativity in nurses’ responses^6^ and staff avoidance of the patient.^7^ Patients with a personality disorder diagnosis are acutely aware of pejorative staff attitudes and feel rejected and disbelieved by clinicians.^8^

Improvement in attitudes has been accomplished through the use of specific psychological models to structure personality disorder training.^9–11^ Mentalization-Based Treatment (MBT)^12^ is an effective treatment fo BPD. It has its roots in attachment theory^13^ and is popular in the UK.

Mentalizing relates to awareness of one's own mental state and the mental states of those around us and treatment of BPD focuses on promoting and recovering mentalizing capacities.^14,15^ Working through MBT requires clinicians to have awareness of ‘non-mentalizing modes’ or ‘vulnerabilities’ that impair mentalizing. Supplementary Box 1, available at http://dx.doi.org/10.1192/bjb.2021.50, shows examples of key mentalizing vulnerabilities.

Recent studies have used MBT in teaching aimed at changing staff attitudes towards personality disorder. Welstead et al^16^ found that a 2-day skills training in MBT had a small effect in improving staff attitudes (Cohen's *D* = 0.2). In a pilot study Polnay et al^17^ reported that teaching trainee psychiatrists mentalizing skills suggested improved attitudes, but the sample (*n* = 16) was small.

Trainees in psychiatry are in the front-line of services and thus key in improving the experience of patients with a BPD diagnosis. Yet trainees often have limited experience and training in managing personality disorder. Given the pejorative attitudes towards patients with personality disorder among psychiatrists, the lack of formal training associated with poorer attitudes and the centrality of psychiatrists’ views for patient management, it seems crucial to find ways to improve attitudes. This is imperative, given that these trainees will be leading and shaping clinical teams for the next 20 to 30 years.

The objective of our study was to evaluate whether a brief teaching in personality disorder using MBT could improve attitudes among trainee psychiatrists. MBT was chosen as a model to structure the teaching, since it establishes a clear framework for treatment and the management of the clinical encounter with people diagnosed with BPD.

## Method

The study population was the core and specialty (senior) trainees on the Bart's and Royal London psychiatry programme for the years 2011–2017. All trainees (*n* = 65) attending the annual lecture were invited to participate. There were no exclusion criteria. Verbal informed consent was obtained from all participants, witnessed and formally recorded. Ethics approval was obtained from the East London NHS Foundation Trust Ethics Committee.

Trainees completed the 35-item version of the Attitudes to Personality Disorder Questionnaire ( APDQ)^18^ before and after the teaching. (A previous factor analysis indicated that 2 of the original 37 APDQ items had a loading under 0.5, meaning that they did not correlate sufficiently with any subscale score to merit their inclusion in the questionnaire^18^). A score between 1 and 6 was awarded on the basis of each response (mirrored for questions assessing assent to a negative trait), scores were added and averaged for each domain. Each average score was then added up to make the total APDQ score. Missing responses to individual statements were assigned the modal response from the whole group for that statement. No questionnaire had more than four responses missing.

### Description of training module

As part of their Royal College of Psychiatrists membership (MRCPsych) training, core trainees received two 3 h training sessions. The first consisted of a lecture on personality disorder in general, emphasising BPD. The second was on MBT theory and technique as applied more specifically to BPD, covering the following:

- an understanding of the development of mentalizing within attachment relationships
- an understanding of the factors that promote and hinder accurate mentalizing
- linking BPD presenting symptoms in the accident and emergency department (A&E) and modes of non-mentalizing (psychic equivalence, pretend mode, teleological thinking)
- discussion of examples in which mentalizing is impaired
- the use of an MBT approach in the acute setting, together with taking a ‘not knowing’ stance
- techniques potentially helpful in promoting a mentalizing encounter (e.g. clarifying intentions, ostensive cues, rewinding, recognising and responding to non-mentalizing modes).

Specialty trainees received only the MBT training session.

An introductory teaching on MBT theory was followed by presenting an audio recorded dialogue (Box 1) between an assessing doctor in A&E and a patient presenting to the psychiatric team following an episode of crisis and self-harm. Trainees were invited to continue the dialogue in a role-play by taking the place of the doctor, with one of the teachers playing the patient. The patient role necessitated statements demonstrating mentalizing vulnerabilities. In this way, the training was intended to be highly applicable to clinical practice and to allow for live testing of the lectures and practising new skills, with direct feedback from the facilitators on the employment of MBT-based techniques.

Box 1 Dialogue in the accident and emergency department

Hello, my name's Dr Jones and I've been asked by James the psychiatry nurse to come and see you.

**OK.**

Can I start by asking why you came here tonight?

**Why I'm here?**

Yes, I understand from James that you cut yourself earlier today. I'd just like you to tell me what happened.

**Well, I took a razor blade from my boyfriend's razor and I cut into my wrist three times. I've got it bandaged now and I'm absolutely fine.**

OK. I'd like to know why it happened? What was going on for you at the time?

**(sighs) I don't want to have to go through all this again, didn't I say the right thing to the other man I spoke to?**

It isn't about right or wrong, I'd just like to understand what was going on for you at the time.

**(sighs) Well, I went out last night with my boyfriend and some of his friends, it was his birthday. It got late, I had drunk quite a lot and I lost him for a bit, and then suddenly I found him, talking to his ex. They had bumped into each other he said, but there's no such thing as a coincidence, it was obvious he still loves her and when I asked him he said no, but I know that he does. I went into one in front of his mates and then I left. I turned off my phone, I went home and I cut myself, so you see? You think I'm a cliché.**

No, I'm not thinking that. When you cut yourself, what was your intention?

**Well, I wasn't trying to kill myself, if that's what you're suggesting.**

Then why did you do it?

**Oh God! This is ridiculous, you're supposed to be helping me.**

I am trying to help.

**Well, you haven't helped, so you're not really interested are you? You haven't admitted me, you haven't given me any medication. You doctors, you're all the same, you just wait and see what happens. I'm leaving.**

[Trainees were then invited to take the part of the clinician and try to use mentalizing skills to facilitate the dialogue further]

## Results

Demographic information for our participants is given in Table 1. Relatively equal numbers of males and females attended the first session (34 males, 28 females). No demographic variables predicted a decreased likelihood of attending the second session.

**Table 1:** Demographics of the study participants^a^

	Attended both (*n* = 49)	Attended first only (*n* = 16)	*P*
Gender, *n*			n.s.
Male	24	10	
Female	25	3	
Missing	0	3	
Age, years: *n*			n.s.
21–30	12	5	
31–40	27	6	
41–50	8	2	
Missing	2	3	
Ethnicity, *n*			n.s.
White	29	5	
Black	2	3	
Asian	13	5	
Other	1	0	
Missing	0	3	
UK trained, *n*			n.s.
Yes	27	10	
No	19	3	
Missing	3	3	
Time in psychiatry, years: *n*			n.s.
<2	11	3	
2–4	11	6	
5–6	16	1	
7–8	8	2	
>9	3	1	
Missing	0	3	
Level, *n*			n.s.
Core trainee	21	8	
Speciality trainee	28	5	
Missing	0	3	
Previous personality disorder training, *n*			n.s.
Yes	12	3	
No	37	10	
Missing	0	3	

n.s., not significant.

a. Chi-squared tests were done to compare drop-out rates on gender, age, ethnicity, UK training, years in psychiatry, level attained and receipt of personality disorder training in the past.

Among those who attended both sessions (*n* = 49), the mean APDQ score before teaching was 19.51 (95% CI 18.91–20.11) and after teaching it was 20.39 (95% CI 19.75–21.02). There was a significant improvement on composite scores on the questionnaire (Wilcoxon signed-rank test *Z* = 3.961, *P* < 0.001, *r* = 0.40). In a second analysis, we took the pre-teaching score of the 16 participants who completed only the first teaching session, assigned it as their post-teaching score and analysed them together with the 49 participants who attended both sessions. This assumes that questionnaire scores for those who dropped out would neither have improved nor deteriorated from pre- to post- teaching. A Wilcoxon signed-rank test indicated that there remained a significant improvement on composite questionnaire scores, albeit with a slightly reduced effect size (*n* = 65, *Z* = 3.961, *P* = 0.001, *r* = 0.35). Both *r* = 0.35 and *r* = 0.40 are small to moderate effects.^19^

Table 2 presents pre- and post-teaching subscale scores of the APDQ. According to additional signed-rank tests for the five subscales, there were significant improvements in purpose (*P* = 0.001) and enthusiasm (*P* < 0.001). We saw trends towards improvements in enjoyment (*P* = 0.072) and security (*P* = 0.074). There was no significant change on the acceptance subscale, but pre-teaching scores were highest in this domain, and so there was less room for improvement.

**Table 2:** Pre- and post-teaching subscale scores on the Attitudes to Personality Disorder Questionnaire (APDQ)

	All pre-teaching scores (*n* = 65)		Post-teaching score with first measure carried forward for 16 ‘drop-outs’ (*n* = 65)		Pre-teaching score with 16 ‘drop-outs’ excluded (*n* = 49)		Post-teaching score with 16 ‘drop-outs’ excluded (*n* = 49)	
	Mean	s.d.	Mean	s.d.	Mean	s.d.	Mean	s.d.
Total APQD	19.32	2.15	19.98	2.33	19.52	2.16	20.42	2.25
Enjoyment	3.07	0.59	3.14	0.62	3.19	0.59	3.30	0.60
Security	4.29	0.50	4.35	0.55	4.31	0.48	4.39	0.53
Acceptance	4.57	0.55	4.60	0.57	4.58	0.51	4.64	0.55
Purpose	4.17	0.67	4.37	0.67	4.18	0.63	4.48	0.63
Enthusiasm	3.22	0.75	3.52	0.68	3.25	0.78	3.61	0.67

We describe one of the role-plays in supplementary Box 2. Supplementary Box 3 gives some examples captured from the role-plays with trainees that exemplified their mentalizing skills. Trainees did not demonstrate any examples of interventions for the pretend mode. This is consistent with the clinical finding that pretend mode is the most difficult mentalizing vulnerability to both detect and effectively respond to.

## Discussion

The analysis suggests that the teaching gave rise to a small to moderate positive change in trainee psychiatrists’ attitude towards people with a BPD diagnosis. Observation of the trainees’ interventions in the role-plays and in supplementary Box 3 indicate that the participants were able to implement basic MBT techniques to help with mentalizing.

This finding is significant for a range of reasons. Persons with BPD have identified a desire for human interaction and feelings of loneliness as a main driver for attending A&E following suicidal ideation or self-harm.^20^ Negative experiences of emergency hospital care following self-harm may increase future self-harm.^21^ Both of these findings underline the importance of the attitude of the treating staff towards persons with a diagnosis of personality disorder.

### Links to previous research

This study adds to a growing body of research^9–11^ indicating that psychological models are effective vehicles for teaching mental health professionals in personality disorder and improving attitudes. Our study's higher number of participants (*n* = 49) confirms Polnay et al's^17^ speculation that an MBT-informed personality disorder training is effective at shifting attitudes about personality disorder, and with a reasonable effect size. Our study also extends Welstead et al's^16^ finding for attitude change in doctors: we found a larger effect size for a homogeneous group of trainee psychiatrists and no evidence of a ‘ceiling effect’, in other words that doctors’ attitudes had less room to improve compared with baseline.

Ring & Lawn's review of patient and clinician perspectives of stigma and BPD found that stigma related to a mutual feeling of powerlessness and to clinicians’ preconceptions about patients and lack of sufficient empathy.^22^ It recommended a multifaceted education strategy that provides clinicians with the necessary empathy, tools, skills and attitudes to work with patients with BPD, as attempted in our training.

### Strengths and limitations

A strength of the current study is the convenience sampling within an established training course. This methodology minimises the effect of participant self-selection that has limited the generalisability of previous studies. When we retested duplicating the baseline scores for non-attendees, we found a minimal drop in effect size, which gives assurance against the positive result being affected by a selection bias.

There are a number of limitations to the study, including the use of an instrument with outdated terminology (e.g. ‘PD patients’) and limited consideration of the increasing methodological and evidence-based use of simulation in training^23,24^ and the use of a script that was insufficiently nuanced. The teacher continuing the scenario in the role of the patient may also have limited the ‘experience-near’ didactic approach (however, trainees appeared fully engaged in the role-plays, possibly owing to the strength of the interpersonal dynamics generated by the scenario, and the teacher was able to feed back on his responses ‘being’ the patient in reaction to the trainee's interventions). Although the scenario was audio recorded, a limitation on reporting of the role-play is that it was not recorded but notes taken instead. A significant limitation is the lack of involvement of people with BPD when we planned the teaching and evaluation.

An additional limitation is the lack of follow-up to examine whether changes in attitude persist. Also unclear is whether an attitude change in fact makes a difference to the experience of patients diagnosed with personality disorder, or leads to more favourable outcomes.

### Clinical implications

MBT-informed teachings in BPD contribute usefully to the clinical training of psychiatrists. MBT is one of a number of evidence-based treatments for personality disorder (e.g. dialectical behavioural therapy,^25^ schema-focused therapy,^26^ cognitive analytic therapy^27^ and transference-focused therapy^28^), all of which have contributions to make in the challenge of the routine psychiatric encounter with patients with personality disorder.^29,30^

These trainings can be incorporated into routine training of psychiatrists (e.g. in preparation for the MRCPsych) as they are resource light (in our case two 3 h sessions). Given their modular self-standing nature, they can easily be delivered by remote teaching and thus be a valuable resource to services lacking expertise in management of personality disorder.

In line with research suggesting that (a) teaching participants clinical skills for use in their work, (b) communicating a psychological model to participants and (c) co-production with people with personal experience of a personality disorder diagnosis all improve the effectiveness of training interventions addressing attitudes towards personality disorder,^31^ we plan that our ongoing trainings for psychiatric trainees (a) will be ‘experience-near’ regarding context (e.g. management of personality disorder in A&E) and teaching delivery (simulations, role-play) and will include a skills component, (b) will use a clear operationalised model such as MBT or transference-focused therapy and (c) will incorporate co-production with people with personal experience of a personality disorder diagnosis.

## About the authors

**Tennyson Lee** is a consultant psychiatrist in medical psychotherapy with DeanCross Personality Disorder Service, East London NHS Foundation Trust, and co-director of the Centre for the Understanding of Personality Disorder, London, UK. **Patrick Grove** is a Principle Clinical Psychologist, Luton CMHTs, East London NHS Foundation Trust, Luton, UK. **Chris Garrett**, is a Consultant in diabetes psychiatry, Bart's Health and East London Foundation Trust, London, UK. **Thomas Whitehurst** is a Clinical Research Fellow, Imperial College, London, UK. **Orestis Kanter-Bax**- is a Senior Trainee in Adult Psychiatry & Medical Psychotherapy, Psychotherapy Services, Essex Partnership University NHS Foundation Trust, Southend, UK. **Kamaldeep Bhui** is co-director of the Centre for the Understanding of Personality Disorder (CUSP), London, and Professor of Psychiatry in the Department of Psychiatry, Warneford Hospital, University of Oxford, UK.

## Data Availability

The data that support the findings of this study are available from the corresponding author on reasonable request.

## Supplementary material

For supplementary material accompanying this paper visit http://doi.org/10.1192/bjb.2021.50. click here to view supplementary material
