# Supplementary material for: Teaching trainee psychiatrists a Mentalization-Based Treatment approach to personality disorder: effect on attitudes
Source: BJPsych Bull. 2022 Oct;46(5):298–302. doi: 10.1192/bjb.2021.50 (PMC9768496; doi:10.1192/bjb.2021.50)
Supplement: Supplementary file 1 [file S2056469421000504sup.zip › S2056469421000504sup001.docx]

**Role play, one example (supplementary material)**

The role play below follows from the end part of the scenario setting in Box 1 and we include mentalizing aspects in italics.

Dr: I'd really like you to stay

Patient: what's the point – you're only going to judge me

Dr: I will try not to

Patient: you will! *(Psychic equivalence)*

Dr: (Here the trainee paused a while and then said:)

You know, I'm asking why you felt the need to harm yourself (*Transparence regarding own mental state, modelling curiosity)*. Can you help me to understand? *(collaboration) (inviting patient to mentalize herself)*

(The patient paused and then disclosed how small and unworthy she had felt at the point when she had seen her boyfriend talking to his ex-girlfriend)

The role play demonstrates the ability of the trainee to implement a number of key MBT skills. While he may have had some of this ability pre-training, the role play allowed both himself and his fellow trainees a more conscious formalising of helpful ways to interact with people diagnosed with BPD . The teacher doing the role play was also able to feedback his experience of being the patient, saying *I had to work quite hard to maintain my hostility.* This allowed for discussion of what specific interventions by the trainee had led to this – ie this method of teaching allows for exploration of microsecond level mental state shift responses to interventions.
