# Supplementary material for: Teaching trainee psychiatrists a Mentalization-Based Treatment approach to personality disorder: effect on attitudes
Source: BJPsych Bull. 2022 Oct;46(5):298–302. doi: 10.1192/bjb.2021.50 (PMC9768496; doi:10.1192/bjb.2021.50)
Supplement: Supplementary file 1 [file S2056469421000504sup.zip › S2056469421000504sup002.docx]

**Box 3: Useful phrases used by trainees to try to promote and recover mentalizing (online supplementary material)**

“I’ll try to explain where I’m coming from,” (Skill: clarifying own intentions).

“Did I say something that made you want to leave?” (Skill: Taking “Not Knowing” stance).

“Can we pause for a second? I’m really concerned about what you’re saying, seems so important and I want to make sure I understand it properly,” (Skills: Empathic validation, rewinding).

“I’d like it we can talk it through first, so we can try to make the best decision we can,” (Skill: managing teleological thinking).

“I want to do something to help and we can admit you, but I’m concerned that doing that might backfire, you’ve said just now you hated the hospital. Can we think that over?” (Skill: managing teleological thinking).

‘I can see you’re distressed’ (Skill: managing psychic equivalence through empathic validation of subjective experience)

‘Can you help me understand how you got there?’ (Skill: managing psychic equivalence through being curious, asking in an interested way how patient reached that conclusion)
