# Supplementary material for: Teaching trainee psychiatrists a Mentalization-Based Treatment approach to personality disorder: effect on attitudes
Source: BJPsych Bull. 2022 Oct;46(5):298–302. doi: 10.1192/bjb.2021.50 (PMC9768496; doi:10.1192/bjb.2021.50)
Supplement: Supplementary file 1 [file S2056469421000504sup.zip › S2056469421000504sup003.docx]

**Box 1: Recognising mentalizing vulnerabilities (online supplementary material)**

**Psychic Equivalence**: An experience where reality is almost "too real". In this state of mind, there is no ‘as if’, the person claims privileged access to their own and other's mental states, without appropriate questioning, and affect tends towards paranoid hostility.

Examples might include:

- What's the point in talking to you? Doctors never listen.
- No-one cares, no-one ever has, and no-one is going to help me now.
- The only reason you're admitting me is to protect yourself.
- You’ve never been through this, there’s no point in talking to you

**Pretend Mode**: Mental states have little relation to reality or current situations. Whilst the person's talk may refer to mental states, these can be contradictory and don't further understanding.

Examples might include:

- "Yes, he was abusive to me for years but one day I just let it go. Forgive and forget."
- "I don't care if you let me go or admit me".
- "I might have hurt myself because of what happened, it could been the weather, or maybe someone looked at me funny."

**Teleological Thinking**: Mental states only exist when evidenced in action, where ‘actions speak louder than words’

Examples might include:

- "I had to harm myself to know how I felt".
- "I couldn't have been that upset, I didn't take an overdose".
- "I know he doesn't care, otherwise he would have picked up the phone".
